# Quantification of collective signalling in time-lapse microscopy images

**DOI:** 10.1515/mim-2024-0003

**Published:** 2024-06-19

**Authors:** Maciej Dobrzyński, Benjamin Grädel, Paolo Armando Gagliardi, Olivier Pertz

**Affiliations:** 27210 Institute of Cell Biology, University of Bern, Bern, Switzerland; Graduate School for Cellular and Biomedical Sciences, 27210 University of Bern, Bern, Switzerland

**Keywords:** cell signalling, image analysis, time-lapse microscopy, spatial clustering, collective phenomena

## Abstract

Live-cell imaging of fluorescent biosensors has demonstrated that space-time correlations in signalling of cell collectives play an important organisational role in morphogenesis, wound healing, regeneration, and maintaining epithelial homeostasis. Here, we demonstrate how to quantify one such phenomenon, namely apoptosis-induced ERK activity waves in the MCF10A epithelium. We present a protocol that starts from raw time-lapse fluorescence microscopy images and, through a sequence of image manipulations, ends with ARCOS, our computational method to detect and quantify collective signalling. We also describe the same workflow in the interactive napari image viewer to quantify collective phenomena for users without prior programming experience. Our approach can be applied to space-time correlations in cells, cell collectives, or communities of multicellular organisms, in 2D and 3D geometries.

## 1 Introduction

**Figure 1:**
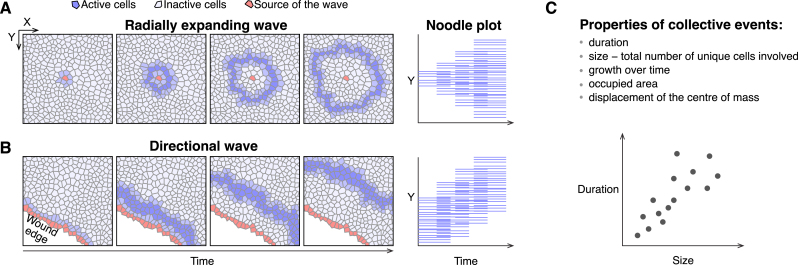
Schematic collective events. (A) Radially expanding waves, as in collective ERK/Akt activity induced by a cell undergoing apoptosis in epithelial tissue. The noodle plot on the right visualises collective events on a space-time plane. Each line corresponds to a single cell when it participates in the event. (B) Directional waves, as in travelling ERK activity waves originating at the edge of a wound. (C) Properties of collective events calculated in our pipeline.

In this tutorial, we describe a computational pipeline to identify apoptosis-induced collective ERK activity waves from fluorescence microscopy time-lapse images of a starved wild-type MCF10A epithelium (Figure 1A). We previously described this biological phenomenon in which apoptotic epithelial cells trigger extracellular signal-regulated kinase (ERK) and AKT serine/threonine kinase (Akt) activity waves that are transmitted radially to healthy neighbouring cells [1]. The waves are mediated within the epithelium through matrix metalloproteinase (MMP) and epidermal growth factor receptor (EGFR) signalling. ERK/Akt waves provide spatial survival signals that locally protect cells around the epithelial injury from apoptosis for 3–4 h. At the cell population level, ERK/Akt waves contribute to epithelial homeostasis (EH) by increasing the overall cell survival in response to perturbations. Disruption of this spatial signalling system prevents a model epithelial tissue from maintaining a barrier function in response to environmental insults.

ERK activity waves have recently been demonstrated to be crucial in the maintenance of epithelial homeostasis [2], [3], acinar morphogenesis [4], osteoblast regeneration [5], cell cycle progression, and the coordination of collective cell migration in wound healing [6], [7] (Figure 1B). However, despite our tutorial focusing on cell signalling, the methodology presented here can be applied to wave phenomena emerging in other systems, such as action potential propagating within individual neurons, chemical, mechanical, and genetic waves in cell collectives, as well as waves in communities of multicellular organisms [8].

As we demonstrate below, our computational pipeline quantifies collective events (CEs) in segmented time-lapse images both, programmatically and through a graphical user interface. The calculated properties of CEs include: duration and size, i.e., the total number of unique cells involved in an event, growth dynamics of individual events, convex hull around events, and displacement of the centre of mass over time (Figure 1C).

## 2 Methods

The human mammary cells MCF10A were stably transfected with a nuclear Histone 2B (H2B) marker for identifying and tracking nuclei, and an ERK-KTR biosensor for measuring ERK activity. The latter reversibly translocates from the nucleus to the cytosol upon ERK activation [9]. Images were acquired for both channels for 25 h at a 5-min interval, resulting in two TIFF stacks, 300 frames each, at 1,024 × 1,024 pixels that cover a field of view of 332.80 × 332.80 μm (Supplementary File 1) [10]. Cells were cultured in starving medium, without external stimulation, which minimises proliferation rate and maintains ERK activity at the basal level, where ERK activity pulses are mainly due to apoptotic-induced waves. For a detailed description of experimental procedures, refer to [8].

**Figure 2:**
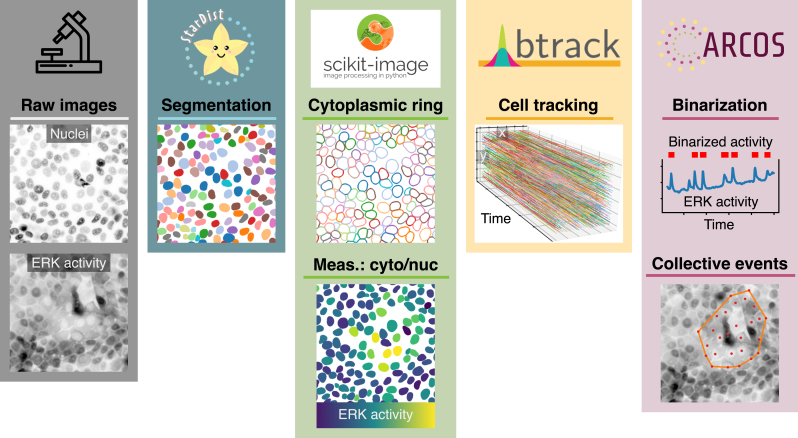
Pipeline overview. Bioimage analysis steps required to identify collective ERK activity waves in the MCF10A epithelium. We use StarDist [20]–[22] to segment the nuclei; scikit-image [23] to manipulate labels and create cytosolic masks to measure fluorescence intensity; btrack [25] to track the nuclei over time; ARCOS [8] to identify and quantify spatio-temporal correlations in ERK activity signalling.

The image analysis pipeline is implemented as a Jupyter notebook [11] with the Python programming language [12] (Figure 2 and Supplementary File 2) [10], [13]. Optional, interactive visualisations of every step are available in the napari image viewer [14]. The core algorithm for Automatic Recognition of COllective Signalling (ARCOS) is implemented in the arcos4py package [15] and extensively described in [8]. Additionally, the analysis relies on several Python libraries that need to be installed, e.g., using the pip install command, and imported before proceeding:

– numpy [16] and pandas [17] for data processing,
– tifffile for reading and writing TIFF image stacks [18],
– joblib for enabling parallel processing on multiple CPU cores [19],
– napari for interactive viewing of time-lapse images [14],
– stardist for image segmentation [20]–[22],
– skimage [23] and an optional package used in napari GUI – pyclesperanto_prototype [24] for manipulating labels obtained from segmentation,
– btrack for tracking objects over time [25],
– matplotlib for plotting the results in the Jupyter notebook [26].

We recommend creating a Python environment using a package manager such as conda or mamba [27]. YAML configuration for the Python environment for macOS, Windows and Linux is provided in Supplementary File 3.

### 2.1 Loading the data

Images, provided as two TIFF stacks with nuclear and ERK activity channels, respectively, can be loaded as NumPy arrays with the imread function from the tifffile package [28]:


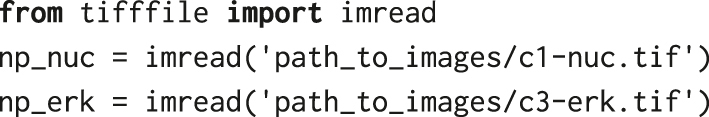


Each stack is stored in the *TYX* order, where *T* corresponds to the time axis, and *YX* are spatial coordinates of the image. To view and interactively browse the stack, we turn to the napari image viewer, which can be accessed programmatically from the notebook. The following code opens a separate viewer window:


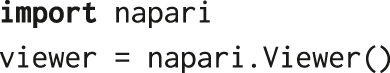


The method viewer.add_image() can be then used to add individual stacks as layers in napari, together with the colour map specification and contrast limits:


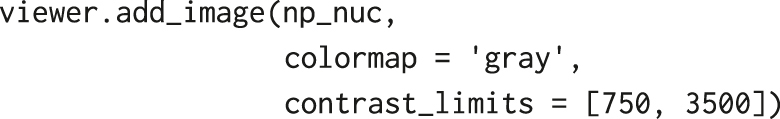


### 2.2 Image segmentation

For identifying nuclei from the H2B channel, we use a Python implementation of star-convex object detection for 2D and 3D images, as described in [20]–[22]. The method is based on a convolutional neural network that, for every pixel, predicts a polygon for the cell instance at that position. The package provides a pretrained model, 2D_versatile_fluo, for 2D images of nuclear fluorescent markers. As a preparation step, we create an instance of a pretrained model:


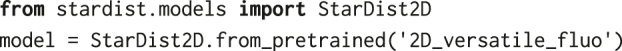


We then pre-allocate a NumPy array of the same size as the image stack, which we will use to store segmentation labels. The input images need to be normalised to the same range as the data used to train the StarDist model, i.e., [0,1]. Therefore, we normalise fluorescence intensities in every frame using normalize from the csbdeep.utils package. Finally, we loop over frames of the H2B channel stack and predict the location of nuclei using StarDist’s model.predict_instances. If no parameters are provided, the prediction step uses a default threshold for the probability, 0.479071, which can be overridden with the prob_thresh parameter.


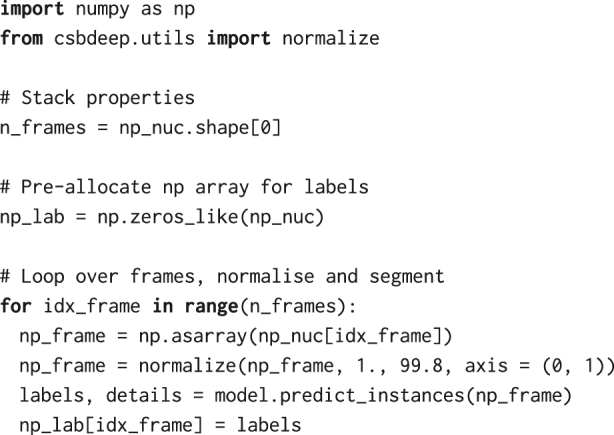


### 2.3 Label manipulation

The NumPy array np_lab resulting from the segmentation is a so-called *labelled mask*. It contains, for every frame, integers denoting object identifiers of nuclei at every pixel location where the StarDist model predicted the nuclei. It will later allow us to extract measurements of individual cells, e.g., fluorescence intensity or area, from the images. However, before proceeding, we process the labels using several functions from the *scikit-image* library for image processing [23].

**Figure 3:**
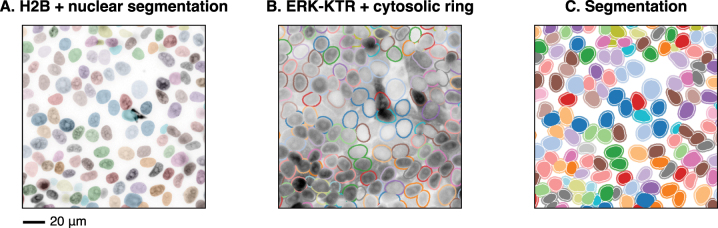
Image segmentation. The result of nuclei segmentation with StarDist (A) and identification of cytosolic rings (B) overlaid on nuclear and ERK channels, respectively. (C) Nuclear and cytosolic ring masks.

First, we remove small labels that correspond to debris or wrong segmentation using the remove_small_objects function from the skimage.morphology utility module of *scikit-image*. To obtain the final nuclear mask, we set the threshold for the minimum area of nuclei (Figure 3A). The filter threshold min_size = 200 removes all objects comprising less than 200 pixels, which corresponds to a round object of diameter ≈16 pixels (5.2 μm).


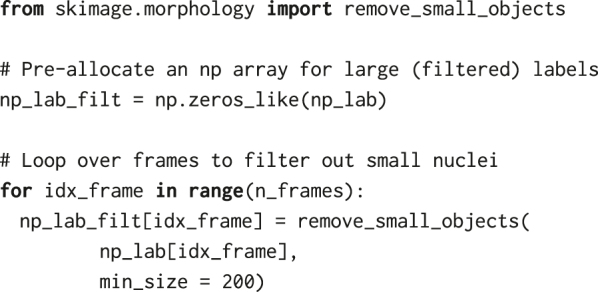


To quantify ERK activity from the ERK-KTR translocation biosensor, we calculate the ratio between the mean cytosolic and nuclear fluorescence intensities, which requires creating an image mask for the cytosol. For every time frame, we perform a series of operations on the existing nuclear mask that create a 4-pixel-wide ring around the nucleus. In the absence of a cytosolic or membrane marker, the ring acts as a proxy for the cytosol. First, we expand the nuclear labels by 6 pixels using expand_labels from skimage.segmentation. Then, we subtract a smaller mask, also obtained from the nuclear mask but only by expanding it by 2 pixels. Rings are 4 pixels wide, with a gap of 2 pixels between the nuclei and rings (Figure 3B).


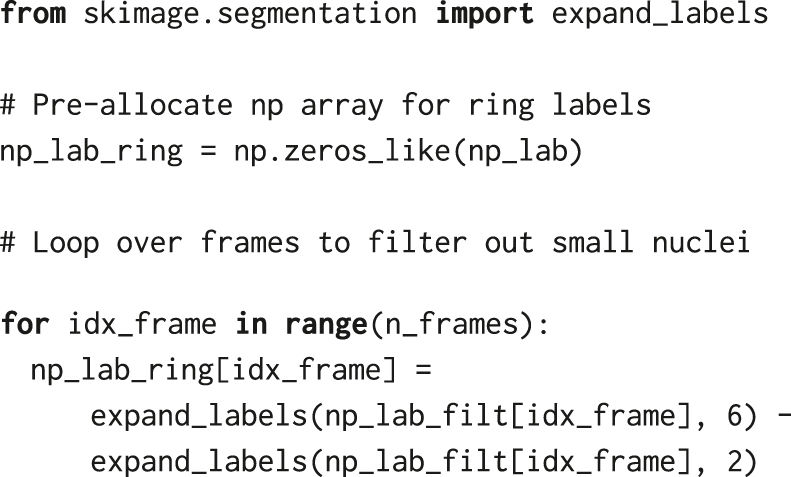


The exact pixel value by which to expand the nucleus and the thickness of the cytosolic ring depends on cell size and density. Importantly, the ring should minimally overlap with neighbouring cells. We recommend verifying the results visually by plotting the masks on raw images, as in Figure 3A and B.

### 2.4 Extracting measurements

Having identified labelled masks for nuclei and cytoplasmic rings (Figure 3C), we can now extract properties from the ERK-KTR channel for every labelled region in the field of view. Again, we loop over frames of np_lab_filt and np_lab_ring arrays, and apply regionprops_table from skimage.measure to extract the mean fluorescence intensity, centroid coordinates, and label identifier that allows us to track the nuclei:


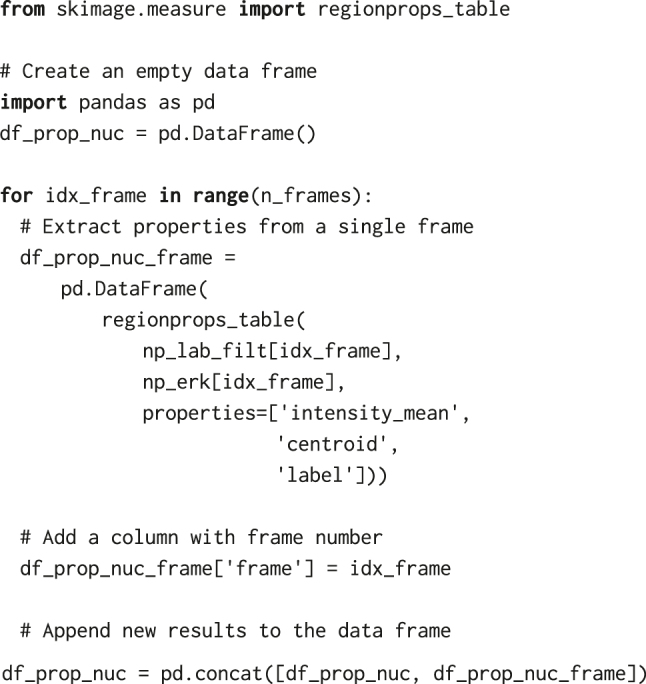


Similar steps, but only with properties = [‘intensity_mean’, ‘label’], yield another pandas data frame, df_prop_cyto, with properties for the cytosolic ring. To obtain a single data frame containing, in a single row, nuclear and cytosolic properties of a cell at a time point, we merge the two tables based on two common key columns: object label ‘obj_id’ and frame number ‘t’ (both integers). To facilitate the merge, we rename the columns in df_prop_nuc and df_prop_cyto generated by regionprops_table, i.e.:

–
label→obj_id ,
–
intensity_mean→intensity_mean_nuc and intensity_mean_cyto for df_prop_nuc and df_prop_cyto, respectively,
–
centroid−0→y and centroid−1→x , which are only present in df_prop_nuc.

**Figure 4:**
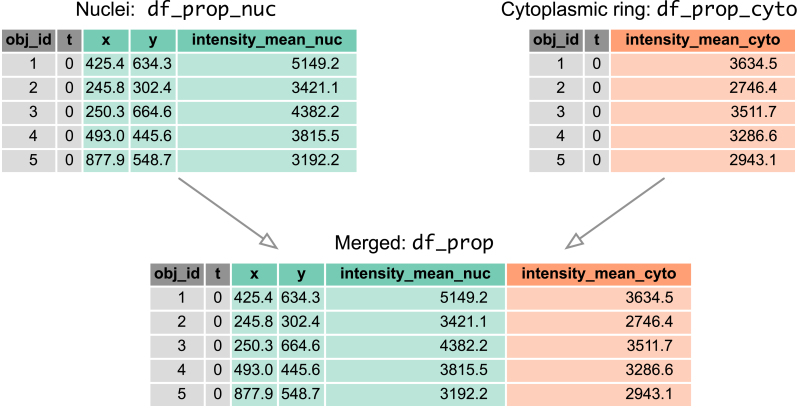
Merging tables. Two pandas data frames containing properties calculated from nuclear and cytoplasmic ring masks, respectively, are merged by two common key columns: object label ‘obj_id’ and frame number ‘t’ (both integers). The resulting ‘df_prop’ contains object label identifiers, positions of nuclear centroids, and nuclear and cytoplasmic fluorescence intensities.

Using the pd.merge function, we obtain a single data frame, df_prop, with object label identifiers, positions of nuclear centroids, and nuclear and cytoplasmic fluorescence intensities, from which we can later calculate the cytoplasmic/nuclear ratio, C/N ERK-KTR (Figure 4).


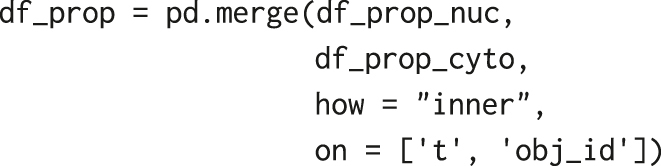


### 2.5 Tracking

To track single cells over time, we turn to another Python library, btrack [25]. It is a commonly used algorithm that uses objects’ positions and properties, e.g., labels’ area, to reconstruct trajectories in crowded environments such as the confluent epithelium discussed here. For the full code required to track the nuclei, we refer the reader to the Jupyter notebook (Supplementary File 2). Configuration parameters and the motion model for particle tracking are stored in a JSON file (Supplementary File 4), and can be loaded using the configure method of the BayesianTracker class. The parameter of interest to us is the update_method, which we set to BayesianUpdates.APPROXIMATE, which calls a faster, approximate algorithm suitable for larger datasets. The associated parameter, max_search_radius, determines the local spatial search radius. We set it to 70 pixels (22.75 μm), which corresponds to the upper limit of a cell diameter and is sufficient to track frame-to-frame cell displacements encountered in our time-lapse (Figure 3A and Supplementary Video 1). A smaller search radius may fail to track moving nuclei, which results in disjointed segments that should otherwise comprise a single track. When max_search_radius is too large, a single track label may be assigned to different objects in subsequent time frames, thus making measurements extracted from such tracks worthless.

The input data for btrack is a NumPy array with labelled nuclei, np_lab_filt, from which *x* & *y* coordinates and the nuclear area are extracted to facilitate tracking. After tracking, btrack outputs a Python list, where each element is a data frame (object of class Tracklet). One such data frame contains a table in long format of a single track, where rows refer to objects in the track at consecutive time points. All those data frames from the list are then merged with the data frame df_prop that contains nuclear and cytoplasmic measurements, using common columns ‘t’ and ‘obj_id’. We thus obtain a data frame df_tracks suitable for the last step of collective event identification with ARCOS. It contains nuclear positions and cytoplasmic/nuclear ERK-KTR ratio measurements in all time frames, with an additional column track_id that holds integer identifiers of tracks/time series. Additionally, btrack provides convenience functions to obtain the data in a format suitable for loading into napari (Supplementary File 2).

**Figure 5:**
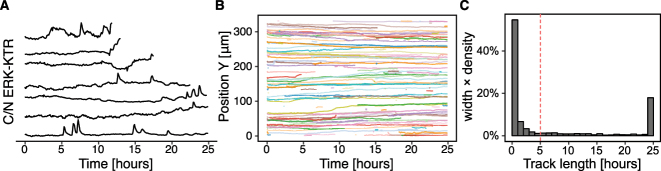
Cell tracking. (A) The result of cell tracking over time with btrack. Each line corresponds to a single cell and its spatio-temporal position. (B) Sample 200 single-cell trajectories projected along the *X* axis. Note several short tracks. (C) Histogram of track lengths. A vertical dashed line at a 5 h mark indicates a cut-off, below which short trajectories are discarded.

Tracking and merging with the measurement table yield single-cell ERK activity time series (ERKATS) (Figure 5A). Several short tracks are still present in the dataset, which may stem from errors in cell segmentation or simply from cells dying during the acquisition (Figure 5B). To perform detrending and binarisation of ERKATS in the next section, we remove time series shorter than 5 h (Figure 5C). This step is required for detrending that applies a long-term median filter with a 2 h window. For a window of that size, time series of at least twice the window length are recommended.

### 2.6 Collective events

**Figure 6:**
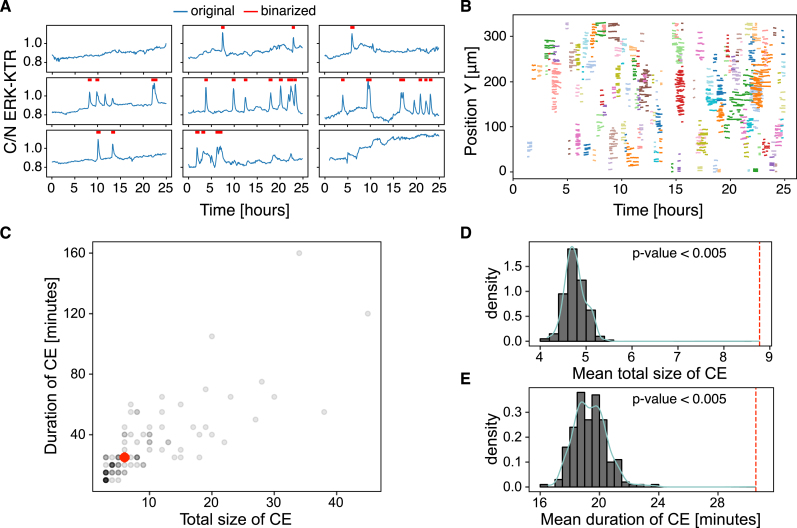
Collective events. (A) Sample detrended and binarised single-cell ERKATS calculated from the cytoplasmic/nuclear ERK-KTR ratio, C/N ERK-KTR. The binarised activity data indicated by red segments is the input for ARCOS. The plot obtained with plotOriginalDetrended from the arcos4py package. (B) Noodle plot visualises CEs identified by ARCOS by projecting them on space-time axes. Each cluster of identically coloured lines corresponds to a single CE, with each line indicating when a single cell participates in the CE. The plot obtained with NoodlePlot from the arcos4py package. (C) Duration versus the mean total number of unique cells in a CE, i.e., “the total size” of a CE. The red dot indicates the median statistics. (D–E) Distributions of statistics calculated from 200 randomisation iterations compared to the observed statistics indicated by the vertical dashed line: the mean total size of CEs (D), and the mean duration of CEs (E). The *p*-value was calculated as the fraction of cases when the statistic was at least as extreme as observed.

Before we identify collective ERK signalling events with ARCOS, we need to further process ERKATS to remove long-term trends in ERK activity and to identify ERK activity periods, which is the required input for our algorithm. Detrending works in two steps: (1) apply a short-range median filter with a 3-time-point window to remove high-frequency oscillations and incidental peaks due to experimental noise, (2) subtract long-term median-smoothed ERKATS from the time series from step 1. Detrending aims to remove long-term trends due to photobleaching of fluorescent probes, or to remove permanently active cells. The subsequent thresholding of the detrended signal may lead to the identification of longer pulses only during their peak activity, thus omitting the leading and the decaying phase of the pulse (Figure 6A). Both detrending and binarisation, can be performed using a convenience method bin_measurements from the arcos4py package that operates on the ARCOS object defined as:


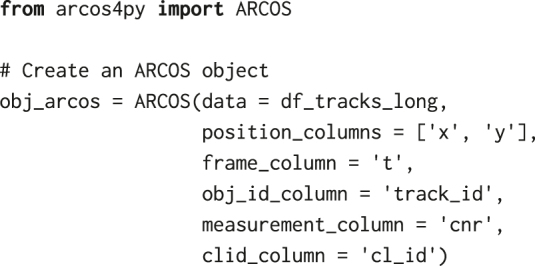


where df_tracks_long is a pandas data frame with single-cell time series obtained from tracking and filtering by length, and the remaining parameters define names of relevant columns, i.e., *x* & *y* positions, frame number, track identifier, measurement of the cytoplasmic/nuclear ratio, and cluster identifier that will be determined in the next steps.

With the following code, and smoothing and thresholding parameters, detrended and binarised ERKATS are added as two additional columns to the input data frame with suffixes resc and bin, respectively:


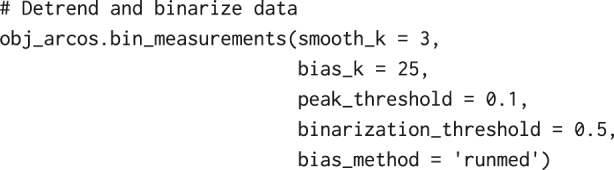


where smoothK and biasK are window lengths in time points of the short- and long-term median filters, respectively; peakThr is a threshold for detecting peaks in a signal rescaled to [0,1]; binThr is a threshold for final signal binarisation; biasMet is a name of the detrending method. We recommend testing several smoothing/thresholding parameters and verifying the results graphically to obtain the best outcome.

At last, collective events (CEs) are detected and tracked from ERKATS with the code below:
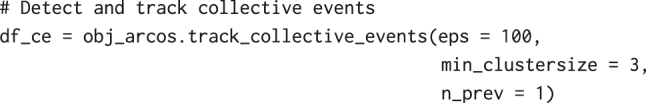


where eps is the search radius in pixels for spatial clustering in individual frames with DBSCAN; minClsz is the minimum number of cells that comprise spatial clusters identified by DBSCAN; nPrev is the number of previous time frames which are used to link spatial clusters in the current time point. The choice of the search radius depends on cell density and the size of clusters of active cells. If set too large, nearby independent CEs are included in a single cluster; too small, and a single CE is identified as several smaller events. A utility function estimate_eps from the arcos4py.tools module helps to estimate eps based on nearest neighbour distances:


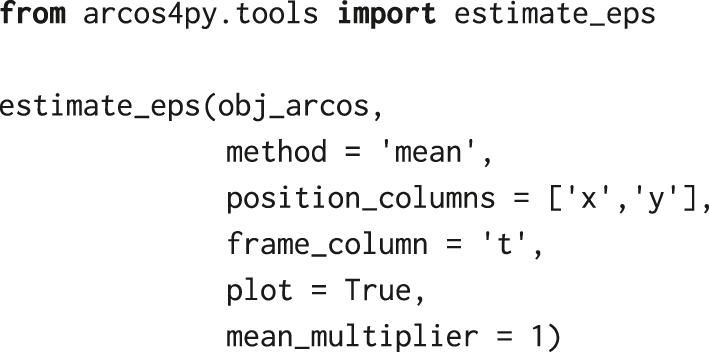


The function returns the plot of sorted nearest neighbour distances and the mean, which for our dataset amounts to 44 pixels (14.3 μm). Considering the variability in cell-cell distances, we assume the search radius eps to be 100 pixels, or 32.5 μm, which is approximately twice the mean nearest neighbour distance. When testing smaller values of eps and after inspecting them against raw ERK activity images in the napari image viewer, we obtained fragmented CEs that should belong to a single event. Conversely, larger eps yielded CEs that were assigned a single identifier but stemmed from several independent events.

The noodle plot is a convenient visualisation of collective events projected onto a 2D space-time plane (Figure 6B). Each trace corresponds to a single cell involved in a CE identified by the algorithm, and CEs are coloured by their identifier. The Supplementary Video 1 visualises identified CEs in the raw time-lapse.

### 2.7 Validation

Statistics such as the mean duration of CEs or the mean total number of unique cells involved in CEs, i.e., “the mean total size”, can be computed from the output of ARCOS (Figure 6C). These observed statistics of CEs can be further tested against a null hypothesis that they do not differ from the statistics of CEs calculated in a system with an equivalent total ERK activity, but with the activation occurring randomly in space and time. The ARCOS package implements 5 randomisation methods to simulate the null hypothesis with varying assumptions. Here, we randomise the time series by shuffling them in space between initial spatial positions of existing time series (cf. Figure S1B in [8]). This method randomises only the spatial component while conserving individual cells’ measurement dynamics and the population-averaged activity over time. By computing the same statistics from the randomised data, we can then calculate the *p*-value, which is the fraction of cases when the statistic was at least as extreme as the observed statistic.

After importing validation module from the arcos4py package, we randomise the time series:


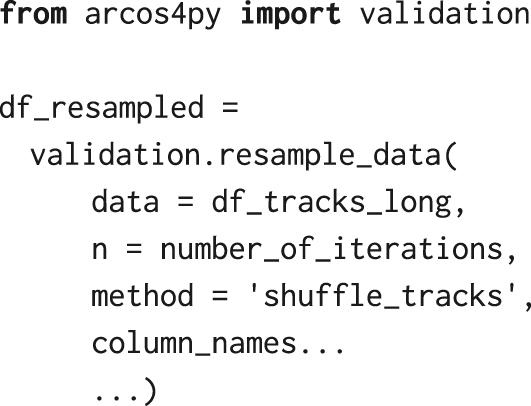


and calculate the statistics from independent randomisation iterations:


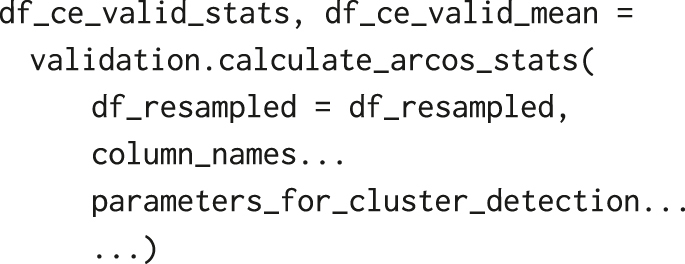


Both functions have the option to execute the calculation in parallel by setting the parameter parallel_processing to True. After repeating the randomisation 200 times, on average, the CEs involved fewer cells and were shorter-lived than the observed average statistics (Figure 6D and E). Since none of the test statistics were at least as extreme as the observed statistic, the *p*-values for the duration and total size were:

(1) $$p=\mathrm{Pr}\left(T\geq t|H_{0}\;\text{is true}\right)=\frac{1+\#\left\{t_{r}\geq t\right\}}{N_{r}+1}\;=\frac{1}{201},$$

where *T* is the test statistic, *t* is the observed value of the test statistic, *N*_*r*_ is the number of independent randomisations from which we obtain the values *t*_*r*_ of the test statistic *T* [29]–[31].

### 2.8 Exporting data

Data can be exported at every stage of the analysis. For example, NumPy arrays with labelled masks can be saved as compressed binary files:


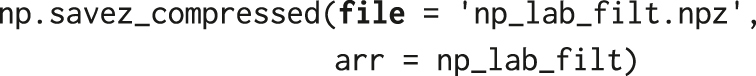


and pandas data frames can be exported to compressed CSV files:


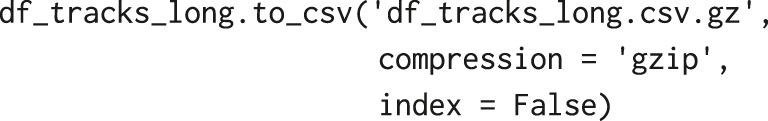


The current napari view with blended visible layers can be saved as an RGB TIFF stack using the napari-timestamper package [32]:


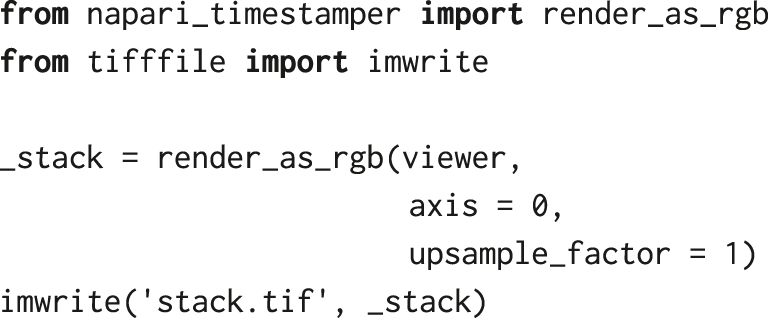


## 3 napari UI

As an alternative to the programmatic approach described so far, the entire workflow can also be run interactively with the napari ARCOS plugin [33]. Here, we outline the steps required to identify collective signalling events from raw microscopy images, and the accompanying screencast demonstrates each step in the UI (Supplementary Video 2).

First, the following plugins must be installed through the napari plugin manager:

1. *stardist-napari*,
2. *btrack*,
3. *napari-assistant*,
4. *napari-segment-blobs-and-things-with-membranes*,
5. *napari-pyclesperanto-assistant*,
6. *napari-skimage-regionprops*,
7. *arcos-gui.*

Installation instructions for individual plugins can be found on the napari hub. Note that it is recommended to install the napari-assistant via one of the plugins that use it as a graphical user interface. Therefore, we recommend installing devbio-napari [34], which bundles many of these plugins.

### 3.1 Pre-processing

We begin the analysis by loading the images into napari. If they are in a standard format, such as TIFF files, no additional plugin must be installed. For other file formats, e.g., NIKON nd2, or Leica lif, specific plugins, such as *napari-aicsimageio*, can be employed.

In the example presented here, TIFF stacks for nuclear and ERK channels, are loaded into napari, and the contrast is adjusted to visualise the individual images properly. The goal is to generate the same two segmentation masks as before, i.e. the ring mask and the nuclear mask, used to measure the fluorescence intensity of the ERK-KTR in the ring and nucleus respectively. As in the Jupyter notebook pipeline, we use StarDist to segment the nuclear channel. Open the *stardist-napari* plugin and confirm that:

1. The correct image is selected,
2. The “Model Type” is set to 2D,
3. The “Versatile” (fluorescent nuclei) model is selected,
4. The axis order is set to *TYX*.

Click on “run” to segment the nuclear channel. Once the plugin has finished processing the image stack, it returns the nuclear segmentation as a labels layer and adds it to the viewer.

To create a cytosolic ring, we use the *napari-assistant*. Said plugin provides a simple interface to set up image processing workflows. The plugins *napari-segment-blobs-and-things-with-membranes* and *napari-pyclesperanto-assistant*, provide functions that can be accessed from within the *napari-assistant*. To use the assistant ecosystem, images must adhere to a standard order of time and space dimensions for inter-plugin communication to work properly. Specifically, image stacks must have the shape *TZYX*, where *T* is the time and *ZYX* are spatial dimensions. Our data comprises 300 time points (frames), 1,024 × 1,024 pixels each. The correct dimension order for the *napari-assistant* is thus “300,1,1024,1024”. So far, all layers in the napari viewer were loaded as *TYX*, which outwardly appears to be the same as a 3D stack with the shape *ZYX*. For the plugin to treat our stack as a time-lapse, an additional, dummy dimension *Z* needs to be inserted. The *napari-assistant* also installs a utility plugin *napari-time-slicer* to perform this conversion. We open a widget called *Convert 3D stack to 2D time-lapse* from the “Tools > Utilities” section and process every layer with it. Thereafter, all the source layers can be discarded.

We can now set up a workflow that will generate the ring mask. We use the following functions in the order as shown below:

1. Process Labels > Expand Labels; 4 pixel,
2. Process Labels > Reduce Labels to Label Edges,
3. Process Labels > Expand Labels; 2 pixel.

The search bar at the top of the plugin window can be used to quickly find the corresponding functions in the napari-assistant. As input for the first function, select the nuclear segmentation; for the second, select the output from the first function, and so on until a ring is generated.

Using the *napari-skimage-regionprops* plugin, we measure the mean intensity for the nuclear mask and the ring mask in the ERK-KTR channel. In the “Tools” sub-menu, select “Measurement tables > Regionprops of all frames”. Choose “Intensity” and “Position”. Run this twice, once for the ring, and once for the nuclear segmentation mask. The output from this plugin gets attached to the respective labels layer.

The *btrack* plugin can be used on the nuclear mask to track our cells. Tracked cells are not required for the analysis with ARCOS; However, they enable more advanced detrending and data analysis down the line. Simply open the plugin and click on “Track”. This will add a “tracks” layer to napari, where we can visually confirm the tracking accuracy.

### 3.2 Detect collective events

Once we have the measurements for nuclear and cytosolic intensity of the ERK-KTR, we can now load the data into ARCOS and proceed from there. Open the *napari ARCOS* plugin, then in the first tab, choose the “Load Data From Layers” option. In the selection box, choose both labels-layers that were used to measure cytosolic and nuclear intensity, and if nuclei were tracked using *btrack*, select the tracks layer in the menu below. Click on “Load Data” and set the names of the columns in the newly opened dialogue. The individual measurements are named after the layers they originate from and what they contain. To calculate the cytosolic to nuclear ratio, in the “Math on first and second Measurement” row choose “Division”, then set the corresponding values in the dialogue box as first and second measurement. Confirm the choices by clicking on “OK” and then select the “ARCOS parameters” tab. Here, set appropriate parameters for your dataset. The epsilon value can be estimated based on the input data. However, in our example, we set it manually. Use the same parameters as outlined in the programmatic approach before. Since we previously converted our *TYX* image stack to a *TZYX* stack by adding a *Z* dimension with size 1, we need ARCOS to also output this dimension order. To achieve this, set the “Output Axis Order” field just below the last ARCOS parameters to “*tzyx*”, then click on “run ARCOS”. A series of layers will be added to the viewer, containing the detected collective events, their convex hulls, as well as which cells were deemed to be active.

The napari ARCOS plugin provides several interactive plots and tools that help with inspecting and analysing data. Additionally, the parameters used for the analysis, as well as the output and statistics, can be exported, to be used for further analysis and visualisation. The current napari view with blended layers can be exported to an RGB TIFF stack or an MP4 video using a convenient plugin, *napari-timestamper* [32].

## 4 Discussion

We described two single-programming-environment workflows to process time-lapse images of fluorescent biosensors to extract dynamic collective signalling events. We applied them to analyse waves of ERK activity that are triggered by apoptotic cells in epithelial cellular communities [1]. These waves are only one of many examples of similar emergent phenomena that have recently been described [35], [36] due to the expansion of fluorescent biosensor technologies and accurate single-cell measurements [37]. We used ARCOS to identify collective events with different sizes, shapes, and durations, in MCF10A, NRK-52E and MDCK cell lines in 2D cell cultures [1], [8] and in 3D MCF10A acini [4]. Other groups applied our method to waves of calcium activity in neutrophils [38] and in blood progenitors of a wild-type lymph gland [39], as well as ERK activity waves in bronchial epithelial cell lines HBE1 and 16HBE [35]. In addition to fluorescence microscopy images of *in vitro* cell cultures, we used ARCOS to identify collective motion in giant honey bee colonies that employ this visual signal to deter predator wasps [8]. The statistics extracted from our analysis can guide the investigators to better interpret the observed phenomena. For instance, when multiple events are occurring at the same time, it is not trivial to distinguish, just by looking at microscopy time-lapses, actual coordinated events from random fluctuation happening close to each other in space and time. By comparing the statistics of measured collective events with a randomised counterpart, it is instead possible to statistically test the hypothesis whether the observed collective events emerge from stochastic fluctuations of signalling activity.

Aside from the application to the discovery of dynamic, collective, emergent behaviour in different biological systems, our method can be further applied to automatise the screening of several experimental conditions. One such example is the identification of molecular mechanisms of collective events’ propagation, which requires high throughput screening with RNAi or small molecule inhibitors. The multicore parallelisation of our pipeline’s code enables scaling up the analysis to high-performance computing (HPC) clusters, further facilitating the analysis of large screening datasets.

Furthermore, clinical research applications that study pathological alterations of collective signalling across multiple conditions could also benefit from our method. We previously observed that two common oncogenic mutations strongly alter single-cell and collective signalling [8]. This suggests that the alteration of collective signalling is a property of cancer, and might drive cancer progression. Our method has the potential to identify changes in collective signalling dynamics, even in disorganised pathological systems where collective signalling events cannot be easily identified by eye.

## Supplementary data

The following supplementary materials accompany this manuscript:

– **Supplementary File 1**: two TIFF image stacks with nuclear and ERK-KTR channels (1.2 GB).
– **Supplementary File 2**: Jupyter notebook with Python code to perform the entire analysis and reproduce all the plots from the manuscript (3.5 MB).
– **Supplementary File 3**: YAML files to configure conda/mamba Python environments for macOS, Windows and Linux.
– **Supplementary File 4**: JSON file with the particle tracking model for btrack.
– **Supplementary Video 1**: an MP4 video of collective ERK activity waves identified with ARCOS and the animated noodle plot (42 MB).
– **Supplementary Video 2**: an MP4 video with a screencast of steps required to perform the analysis in the napari image viewer (112 MB).

Supplementary Files 1–4 were uploaded to Mendeley Data [10] and files 2–4 to GitHub [13] repositories.

## Supplementary Material

Supplementary Material Details
