# Supplementary figures and images for: Quantification of collective signalling in time-lapse microscopy images
Source: Methods Microsc. 2024 Jun 19;1(1):19–30. doi: 10.1515/mim-2024-0003 (PMC11308913; doi:10.1515/mim-2024-0003)

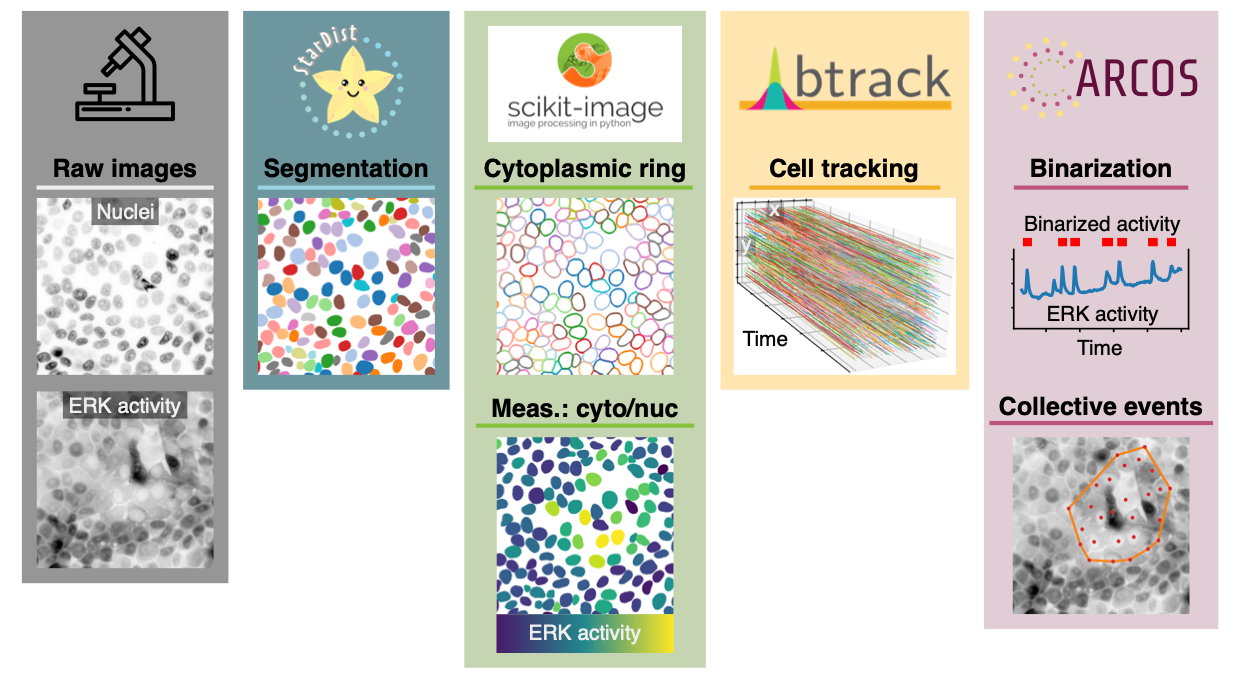

Supplement: Supplementary file 1 — Supplementary Material Details [file j_mim-2024-0003_suppl_001.zip › scripts/extras/napari-flow.png]
